# Rituximab in Relapsing and Progressive Forms of Multiple Sclerosis: A Systematic Review

**DOI:** 10.1371/journal.pone.0066308

**Published:** 2013-07-02

**Authors:** Tamara Castillo-Trivino, Dejana Braithwaite, Peter Bacchetti, Emmanuelle Waubant

**Affiliations:** 1 Multiple Sclerosis Center, Department of Neurology, University of California San Francisco, San Francisco, California, United States of America; 2 Multiple Sclerosis Unit, Department of Neurology, Hospital Universitario Donostia, San Sebastian, Guipuzcoa, Spain; 3 Neurosciences Area, Biodonostia Institute, San Sebastian, Guipuzcoa, Spain; 4 Department of Epidemiology and Biostatistics, University of California San Francisco, San Francisco, California, United States of America; National Institutes of Health, United States of America

## Abstract

**Background:**

Rituximab is an anti-CD20 monoclonal antibody approved for non Hodgkin lymphoma and rheumatoid arthritis. It is being considered for the treatment of MS.

**Objectives:**

To evaluate the efficacy and safety of rituximab for MS treatment.

**Data collection:**

Studies were selected if they were clinical trials, irrespective of the dosage or combination therapies.

**Main results:**

Four studies with a total of 599 patients were included. One assessed the efficacy of rituximab for primary progressive (PP) MS while the other three focused on relapsing-remitting (RR) MS. In the PPMS study, rituximab delayed time to confirmed disease progression (CDP) in pre-planned sub-group analyses. The increase in T2 lesion volume was lower in the rituximab group at week 96 compared with placebo. For the RRMS studies, an open-label phase I study found that rituximab reduced the annualized relapse rate to 0.25 from pre-therapy baseline to week 24, while in the randomized placebo-controlled phase II trial, annualized relapse rates were 0.37 in the rituximab group and 0.84 in the placebo group (p = 0.04) at week 24. Rituximab dramatically reduced the number of gadolinium-enhancing lesions on brain MRI scans for both RRMS studies. Off-label rituximab as an add-on therapy in patients with breakthrough disease on first-line agents was associated with an 88% reduction when comparing the mean number of gadolinium-enhancing lesions prior to and after the treatment. Although frequent adverse events classified as mild or moderate occurred in up to 77% of the patients, there were no grade 4 infusion-related adverse events.

**Author’s conclusion:**

Despite the frequent mild/moderate adverse events related to the drug, rituximab appears overall safe for up to 2 years of therapy and has a substantial impact on the inflammatory disease activity (clinical and/or radiological) of RRMS. The effect of rituximab on disease progression in PPMS appears to be marginal.

## Background

Multiple sclerosis (MS) is a demyelinating disease of the central nervous system and one of the main causes of neurological disability in young people. It is an immune-mediated chronic disorder in which activated T cells have been implicated, causing areas of myelin damage, and oligodendrocyte and axonal loss. These demyelinated areas or plaques accumulate over time and contribute to disability progression.

Clinically, there are different phenotypes of MS. The most common is the relapsing-remitting (RR) form characterized by the development of acute symptoms or relapses of neurological deficits followed by a complete or incomplete recovery, also called remission. Relapse rate and development of new MS lesions on repeat brain MRI scans are the main outcome measures when evaluating efficacy of anti-inflammatory drugs in MS. Progression of disability is another typical outcome measure of drug efficacy. After a variable number of years, most patients develop a secondary progressive course (SP) characterized by a progression of the neurological disability associated or not with superimposed relapses. Other less common types include the primary progressive (PP) form, in which there is slow progression of disability and neurological symptoms without relapses, and the progressive-relapsing (PR) form, in which patients suffer an insidious disability progression from onset with some rare superimposed relapses. In PPMS, clinical trials evaluating the efficacy of promising drugs use progression of disability as the main primary outcome measure, while accumulation of new lesions on repeat brain MRI scans is a secondary outcome measure.

Approved treatments for relapsing forms of MS, such as glatiramer acetate and the three subtypes of interferon beta, have been developed for their effect mostly on T cells in the pathogenesis of MS. However, not all the patients benefit from these therapies as some continue to experience disease activity while compliant to their medication.

Recent evidence has shown that B cells and humoral immunity also play a key role in MS pathogenesis [1], [2]. Memory B cells and plasma cells are found in lesions and cerebrospinal fluid from patients with MS [3], [4], [5]. Thus therapies targeting B cells have been investigated as promising MS treatments in the past few years [6]. Rituximab is a chimeric monoclonal antibody against the CD20 molecule expressed on mature B cells that effectively depletes these circulating B cells [7]. Profound CD20+ B cell depletion is expected to alter B-cell-mediated antigen presentation and resulting activation of T cells, antibody production, and possibly Epstein-Barr virus circulation, a virus that is harbored in B cells and has been implicated as potentially linked to MS pathology.

The use of other monoclonal antibodies for MS, such as natalizumab, have been accompanied by some concerns due to the development of a disabling and often fatal neurological complication, known as progressive multifocal leukoencephalopathy, caused by JC virus infection in the brain [8], [9], [10], but there is limited information about the effect of other monoclonal antibodies in development for MS not only regarding safety, but also efficacy.

## Objectives

The objective of this systematic review is to evaluate the efficacy and safety of rituximab for the treatment of patients with any form of MS. We hypothesized that rituximab is effective in reducing disability progression for patients with any type of MS course, while maintaining a good safety profile.

The secondary hypothesis is that rituximab is effective at reducing the relapse rate in the relapsing forms of MS and reducing the brain MRI activity.

## Methods

### Criteria for Considering Studies for this Review

#### Types of studies

Clinical trials (any kind of clinical trial) including those versus placebo and those comparing rituximab with any other MS therapy alone or in combination. English and Spanish were considered as possible languages.

#### Types of participants

Adult patients (≥18 years old) diagnosed with MS McDonald or Poser criteria [11], [12], [13] affected by any type of MS (RR, SP, PP or PR).

Although rituximab is used to treat neuromyelitis optica, this demyelinating disorder was excluded from the review due to the different pathogenic processes underlying this condition compared to MS.

#### Types of interventions

We considered three types of interventions: i) rituximab alone (comparing disease activity on treatment with baseline activity), ii) rituximab versus placebo or versus any other MS therapy and iii) combination of rituximab with steroids versus placebo or steroids alone.

#### Types of outcome measures

As primary outcomes we considered: i) the proportion of patients with confirmed disability progression at one year, defined as an increase of at least 1 point in the baseline Expanded Disability Status Scale (EDSS) [14] if the baseline is <6 or an increase of 0.5 point in the EDSS if the baseline EDSS is ≥6 and ii) the proportion of patients who withdrew from the study due to major side effects of the drug (causing death or hospitalization of the patient), and patients with grade 4 adverse events (AEs).

As secondary outcomes we considered: i) the annualized relapse rate at the end of the study period and ii) the number of gadolinium-enhancing (Gad-enhancing) lesions on the T1 brain MRI scans and number of new T2 bright lesions on the brain MRI scans.

### Search Methods for Identification of Studies

The following databases were queried to identify relevant articles:

1 MEDLINE (Pubmed)

((“Multiple Sclerosis”[Mesh] OR “Demyelinating Diseases”[Mesh]) AND “rituximab”[Substance Name]) NOT “Neuromyelitis Optica”[Mesh]

2 EMBASE

#1 ‘rituximab’/mj OR rituximab:ti OR mabthera:ab,ti OR rituxan:ab,ti

#2 ‘multiple sclerosis’/de OR ‘multiple sclerosis’/ti OR ‘demyelinating diseases’/mj

#3 ‘controlled clinical trial’/exp OR ‘clinical trial’/de OR ‘clinical study’/de OR ‘controlled study’/de OR randomiz*:ab,ti OR randomis*:ab,ti OR ‘major clinical study’/de OR ‘randomization’/de OR ‘evidence based medicine’/exp OR cochrane:ab,ti

**#4** #1 AND #2 AND #3

3 The Cochrane Library

(rituximab OR mabthera OR rituxan):ti,ab,kw AND “multiple sclerosis” OR “demyelinating disease” OR “demyelinating diseases”:ti,ab, kw

4 ISI Web of Knowledge: BIOSIS & Web of Science

Topic = (rituximab OR mabthera OR rituxan) ANDTopic = (“multiple sclerosis” OR demyelinating disease” OR “demyelinating diseases”) ANDTopic = (radom* OR controlled OR rct OR rcts OR trial OR trials) NOTTitle = (“rheumatoid arthritis” OR optica OR optic)

5 WHO International Clinical Trials Registry Platform (ICTRP)

Rituximab AND multiple sclerosis

6 Current Controlled Trials

Rituximab AND multiple sclerosis

7 Clinical trials.gov

Rituximab AND multiple sclerosisTwo results of Rituximab (MabThera/Rituxan)

### Data Collection

One reader specialized in MS and epidemiology reviewed and selected the articles.

For each trial, information concerning number of participants, interventions, outcomes, follow-up, side effects, randomization and blinding if applicable were collected. For the systematic review and for the evaluation of the quality of the studies, the PRISMA Statement (http://www.prisma-statement.org) [see the PRISMA checklist, Table S1] and the Jadad Score [15] were used as guidelines.

## Results

### Description of the Studies

**Figure 1:**
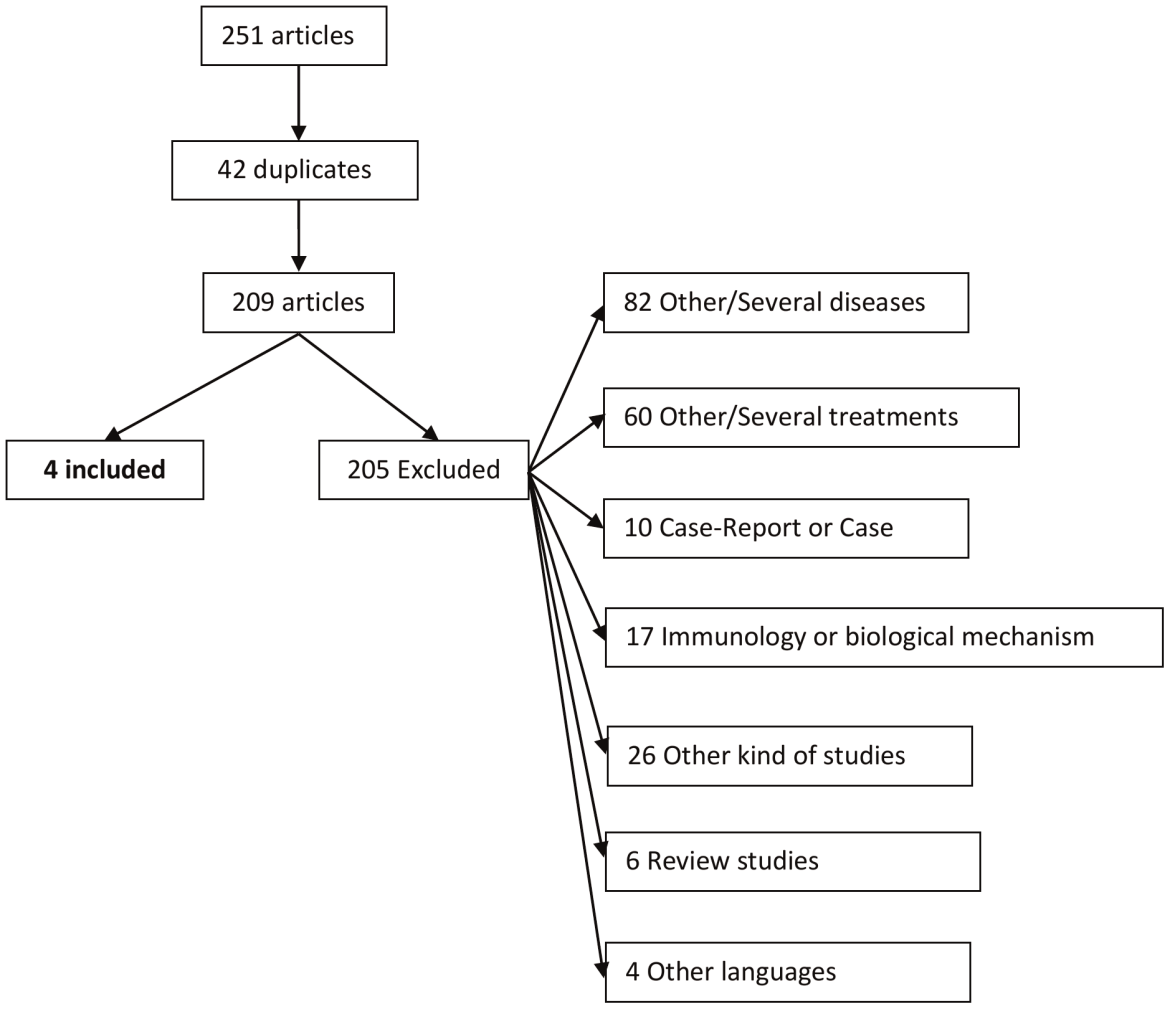
Flow chart with information on the identified and excluded articles.

We identified 251articles and finally four articles were included in this study. The number of included and excluded articles as well as the reasons of exclusion is shown in Figure 1.

From the 209 identified articles (there were 42 duplicates from the 251), 205 were excluded. From these, 82 concerned other diseases such as rheumatoid arthritis, lymphoma or demyelinating polyneuropathies.

Four trials contributed to this review, published between 2008 and 2010. Study 1 [16] is a 72-week, open-label, phase I, multicenter trial of RRMS patients including 26 patients. Study 2 [17] is a 48-week, randomized, double-blind, phase II, multicenter trial including 104 RRMS patients. Study 3 [18] is a 96-weeks, randomized, double-blind, phase II/III, multicenter trial for patients with the primary progressive forms of MS and included 439 patients. Study 4 [19] is a 52-week, phase II, single-center trial including 30 RRMS patients and using rituximab as an add-on therapy to an ongoing first-line disease-modifying therapy (DMT). The characteristics of the included studies are shown in Table 1.

**Table 1:** Characteristics of the included studies.

	Study 1 (Bar-Or 2008)	Study 2 (Hauser 2008)	Study 3 (Hawker 2009)	Study 4 (Naismith 2010)
Phase	I	II	II/III	II
Disease form	RRMS	RRMS	PPMS	RRMS
Patients	18–55 years old	18–55 years old	18–65 years old	18–65 years old
Study period	72-week	48-week	96-week	52-week
Randomization	No	Yes (2∶1)	Yes (2∶1)	No
Placebo-controlled	No	Yes	Yes	No
Double-blind	No	Yes*	Yes*	No
Jadad score	1	5	5	1
Multicenter	Yes	Yes	Yes	No
Patients included	26 (RTX)	104 (69 RTX, 35 PLC)	439 (292 RTX, 147 PLC)	30 (RTX)
Withdrawals or dropouts	Yes	Yes	Yes	Yes (2 patients not considered for study)
% of withdrawals or dropouts	15.4%	15.9% (RTX) 40% (PLC)	17.5% (RTX) 15.6% (PLC)	6.2%
Intention to treat	N/A	Yes	Yes	N/A

RTX = Rituximab, PLC = Placebo.

* Blinding of the patients was questionable due to adverse events.

#### Participants

Studies 1 [16] and 2 [17] recruited adult patients (18–55 years old) with a diagnosis of RRMS with at least one relapse in the preceding year and with an EDSS at entry between 0 and 5.0 (both inclusive).

For study 3 [18] adult patients between 18 and 65 were included, with a diagnosis of PPMS and a disease duration ≥1 year. Baseline EDSS was between 2.0 and 6.5, inclusive, with a functional system scale score of ≥2 for the pyramidal system or gait impairment due to lower extremity dysfunction and presence of IgG oligoclonal bands or elevated CSF IgG or both.

Study 4 [19] is an add-on therapy study that included adult patients aged 18–65 years old diagnosed with RRMS, with a baseline EDSS ≤6.5, treated with an injectable first-line disease-modifying therapy (DMT) for at least 6 months, with breakthrough disease defined as having a clinical relapse in the prior 18 months while taking the DMT, and at least one Gad enhancing lesion on any of 3 monthly pretreatment brain MRI scans.

Patients received brain MRI scans with and without gadolinium at baseline for the four studies and at weeks 4,8,12,24,36,48,60 and 72 for study 1 [16], at weeks 4,12,16,20,24,28,36 and 48 for study 2 [17], at weeks 4,48 and 96 for study 3 [18] and at weeks −8, −4, 12, 16 and 20 for study 4 [19].

Exclusion criteria for study 2 [17] included neuromyelitis optica, PPMS, SPMS or progressive relapsing forms, relapse within 30 days, prior treatment with cyclophosphamide or mitoxantrone within 12 months, systemic corticosteroid therapy within 30 days, treatment with interferon beta, glatiramer acetate, natalizumab, plasmapheresis or intravenous immune globulin within 60 days or non-lymphocyte-depleting immunosuppressive therapies within 90 days. For study 3 [18] the exclusion criteria included history of MS exacerbation or neuromyelitis optica, history of myelopathy or neurodegenerative central nervous system conditions, systemic autoimmune disorders, recurrent or chronic infections, recent treatment with immunomodulating or immunosuppressant therapies and metabolic, hematologic or immunologic laboratory abnormalities. For study 4 [19] patients with other medical illness, aspartate aminotransferase, alanine aminotransferase or creatinine twice normal upper limit, prior use of a major immunosuppressive agent (cyclophosphamide, mitoxantrone, cladribine, natalizumab or other monoclonal therapeutics) or the use of methotrexate or azathioprine within 6 months were excluded. Exclusion criteria were not specified for study 1 [16].

#### Interventions

Study 1 [16] was an open-label trial in which patients received 1,000 mg of IV rituximab on days 1 and 15 and a repeat course on weeks 24 and 26. In studies 2 [17] and 3 [18] patients were randomly assigned 2∶1 to rituximab (same regimen than for study 1) or placebo at weeks 0,2 for study 2 and at weeks 0,2,24,26,48,50,72,74 for study 3. Study 4 [19] was an add-on therapy, and subjects continued their ongoing first-line DMT throughout the study and rituximab was added at weeks 1,2,3 and 4. The dose was 375 mg/m^2^ IV weekly x 4.

Studies 1 [16], 2 [17] and 4 [19] administered premedication (acetaminophen and diphenhydramine hydrochloride) to prevent adverse events before rituximab infusion.

#### Primary and secondary outcomes

The primary outcome for study 1 [16] was the safety of rituximab, determined by adverse events (AEs) and serious AEs, including worsening MS, number and severity of infusion-associated events (defined as those reported during or within 24 hours of an infusion), number and severity of infectious AEs, any clinically significant changes in laboratory or vital sign measurements, the incidence of human anti-chimeric antibodies (HACA) and the total number of Gad-enhancing T1 lesions over the 72-week trial.

For study 2 [17], the primary outcome was the cumulative number of Gad-enhancing lesions on T1-weighted MRI brain scans at weeks 12, 16, 20 and 24.

In study 3 [18] the primary outcome was the time to confirmed disease progression (CDP), defined as a sustained EDSS increase of ≥1 point from baseline EDSS if the baseline EDSS was between 2.0 and 5.5 or an EDSS increase of ≥0.5 points if the baseline EDSS was >5.5 points (if change not attributable to another etiology) sustained for ≥12 weeks.

Study 4 [19] considered the reduction in the sum of Gad-enhancing lesions on T1 MRI brain scans at weeks 12, 16 and 20, compared to the MRI scans obtained at −8, −4 and 0 weeks.

The secondary outcomes for study 1 [16] were proportion of patients experiencing a confirmed relapse and number of relapses per patient during the study, change from baseline in the total number of Gad-enhancing T1 lesions, total number of new T2 lesions and cumulative volume of T2 brain lesions. Finally another outcome included the CD19+ lymphocyte counts to evaluate the pharmacokinetic and pharmacodynamic profile of rituximab.

In study 2 [17], the secondary outcomes were the proportion of patients with relapses, the annualized relapse rate, the total number of new Gad-enhancing lesions on T1-weighted brain MRI scans at weeks 12,16,20 and 24 and the change from the baseline lesion volume on T2-weighted MRI scans.

In study 3 [18], the secondary outcomes included change from baseline to week 96 in the volume of T2 lesions and change in brain volume (brain parenchymal fraction) on brain MRI scans. Exploratory outcomes included time to CDP sustained for ≥24 weeks, change in Multiple Sclerosis Functional Composite (MSFC) summary and component scale scores, CD19+ B-cells counts and immunoglobulin levels and pre-specified subgroup analysis by age, gender, race/ethnicity, use of prior therapy, EDSS at baseline, duration since MS symptom onset and presence of Gad-enhancing lesions on brain MRI at baseline.

Finally, in study 4 [19], Multiple Sclerosis Functional Composite Scale (MSFC) was performed and mean MSFC at weeks −4, 0 was compared to mean MSFC at weeks 24, 28 and 32. Change in EDSS was also explored comparing EDSS at week 32 to baseline. The correlation between CSF B and T cell counts and MRI response and the impact of rituximab on neutralizing antibodies to interferon beta were also explored. Finally, adverse events were recorded.

For both study 2 [17] and 3 [18] there was a neurologist clinically evaluating the patient, blinded to treatment assignment in addition to a treating investigator handling tolerability issues to study drug. For studies 2 [17], 3 [18] and 4 [19], MRI scans were blindly read.

#### Clinical outcomes

Two different clinical outcomes were considered in these studies: disability progression and the relapse frequency or relapse rate.

In studies 1 [16] and 2 [17] disability progression is not provided. In study 3 [18] disability progression is defined as a sustained EDSS increase of ≥1 point from baseline EDSS if the baseline EDSS was between 2.0 and 5.5 points (inclusive) or an EDSS increase of ≥0.5 if baseline EDSS was >5.5 points. This change could not be attributable to other etiology such as fever, concurrent illness, injury, adverse reactions to concurrent medications or recent relapse, and had to be sustained for ≥12 weeks. Study 4 [19] compared baseline EDSS to week 32 and confirmation at week 52. Sustained change in EDSS was defined as in study 3 [18].

Relapses were defined in study 1 [16] as the occurrence of new or worsening neurological symptoms consistent with MS manifestations, evolving over less than 3 months and accompanied by objective neurological worsening, consistent with an increase of at least half a step on the EDSS, two points on one of the appropriate functional system scales (FSSs) or one point on two or more of the appropriate FSSs. The change had to be verified by the investigator and must have affected the selected FSSs. Symptoms had to persist for more than 24 hours and could not be attributable to confounding clinical factors (fever, infection, injury, adverse reactions). Other symptoms that were not accompanied by changes on clinical examination were not considered relapse.

In study 2 [17] relapse was defined as new or recurrent neurologic symptoms consistent with MS that lasted for at least 48 hours, preceded by a relatively stable or improving neurologic status for at least 30 days.

In study 3 [18] relapses were not considered as outcome as all patients had a primary progressive form of the disease (patients do not have relapses by definition). Study 4 [19] did not provide a definition of relapse despite of including RRMS patients and defining breakthrough disease according to clinical relapses and radiological activity. Although this study was not designed to analyze effects on the relapse rate, authors describe the relapse rate at baseline and at the end of the study.

#### MRI outcomes

Study 1 [16], study 2 [17] and study 4 [19] included the number of Gad-enhancing lesions on T1-weighted brain MRI sequences during the study. Study 1 [16] also included the total number of new T2-bright lesions and cumulative volume of T2-bright brain lesions. Study 2 [17] included the total number of new Gad-enhancing lesions on the T1-weigthed sequences. In addition, studies 2 [17], 3 [18] and 4 [19] included the change from the baseline lesion volume on T2-weighted MRI scans. In study 3 [18] change in brain volume was measured by brain parenchymal fraction. Study 4 [19] also included black hole number and volume, and number of T2-bright lesions on brain MRI.

#### Side effects and adverse events

All the studies reported adverse events and classified most of them as infusion- or infection-related. Authors described in the studies the cause of death if any occurred.

### Risk of Bias in Included Studies

Study 1 [16] is a single-arm open-label trial, with the limitations that this may imply. Two other studies [17], [18] are described as randomized, placebo-controlled, double-blind trials. There were a treating investigator who was the safety assessor and made treatment decisions and an examining investigator blinded to treatment and side effects, as well as imaging and laboratory results. The blinding of patients was questionable, as the patients receiving rituximab were more likely to suffer adverse effects related to the infusion. Study 1 [16], study 2 [17] and study 4 [19] used preventive symptomatic medications before the infusions (acetaminophen and diphenhydramine hydrochloride) in order to limit the frequency and the severity of infusion-related side effects.

The reading of the MRI was blinded for studies 2, 3 and 4 [17], [18], [19], and was performed by an independent MRI reading facility for study 2 [17] and study 3 [18].

### Effects of Interventions

#### Primary outcomes

In terms of safety as an outcome, Table 2 summarizes the main adverse events.

**Table 2:** Summary of adverse events seen in the different rituximab studies.

Adverse Events	Study 1 (Bar-Or 2008)	Study 2 (Hauser 2008) PLC RTX	Study 3 (Hawker 2009) PLC RTX	Study 4 (Naismith 2010)
% of patients who completed study	84.6%	60% 84.1%	84.4% 82.5%	93.7%
Any event				
Grade 1 or 2	77%	74.3% 62.3%	61.9% 58.6%	NA
Grade 3	23%	25.7% 30.4%	34.7% 36%	NA
Grade 4 or 5	0%	0% 5.7%	3.4% 4.4%	
Drug related AEs	65.4%	Up to 20%	Up to 20.3%	NA
Infusion-related AEs	65.4%			40.6%
-First infusion	42%	40% 78.3%	23.1% 67.1%	_
-Second infusion	15%	40% 20.3%	15.1% 22.6%	_
All infection-associated AEs	61.5%	71.4% 69.6%	65.3% 68.2%	15.6%
Deaths (number)	0	0 1	2 1	0

RTX = Rituximab, PLC = Placebo, AEs = Adverse events.

In study 1 [16] there were no reported grade 4 AEs. The majority of the 26 patients (77%) experienced mild to moderate (grade 1–2) AEs while six patients reported grade 3 (severe) AEs (including fatigue, tooth fracture, muscle weakness and headache). One of 26 patients discontinued study drug before the end of the study, not receiving the 26 week infusion, due to an infusion-related AE. Infection-associated events were reported by 61.5% of the patients and all were mild-to-moderate (grade 1–2) in severity.

In study 2 [17], 62.3% of the patients in the rituximab group presented grade1–2 AEs (74.3% in the placebo), 30.4% grade 3 AEs (25.7%) and 4.3% grade 4 (none in the placebo group). There were 3 patients who reported grade 4 events in the rituximab group (ischemic coronary-artery syndrome, malignant thyroid neoplasm and symptoms of acute and progressive MS). A total of 5.7% of patients in the placebo group and 4.3% in the rituximab group withdrew from the study because of AEs. There was a death in the rituximab group due to homicide.

In study 3 [18], at week 74 infusion-related reactions were less frequent in the rituximab (4.9%) versus placebo (7.2%) recipients. No grade 4 infusion-related AEs were reported. Infection-related serious AEs (SAEs) were <1% in placebo group and 4.5% in the rituximab treatment. Three patients died during the study. One patient (rituximab group) had a history of brainstem lesions and aspiration and withdrew early from the study. The other two were in the placebo group. One of them died from cardiopulmonary failure during the study and the other contracted pneumonia and died after withdrawing from the study.

In study 4 [19], the infusion-related events led to discontinuation in 2 patients. Eleven patients out of 30 who completed the study had minor reactions that did not preclude additional infusions.

As the main goal of this work was to evaluate the use of rituximab in relapsing and progressive forms of MS, we chose disability as primary outcome, as relapses do not occur in primary progressive forms.

Disability progression as a primary outcome was available for patients in study 3 [18] and study 4 [19]. Although in study 3 rituximab tended to delay the time to CDP at week 96 compared to placebo, with the proportion of patients with CDP of 38.5% and 30.2% respectively (HR = 0.77), the difference was not statistically significant (p = 0.14). In study 4 [19] EDSS improved after rituximab therapy in 7 out of 30 patients, remained stable in 21 and worsened in 2.

#### Secondary outcomes

The effect of rituximab on the relapse rate was one of the secondary outcomes in studies 1 [16] and 2 [17]. In study 1, 80.8% (21/26) of the patients (all of them received rituximab) remained relapse free at week 72. The unadjusted annualized relapse rate was 0.25 from baseline to week 24 and 0.18 from baseline to week 72. In study 2, the proportion of patients with relapses was reduced in the rituximab group (n = 69) at week 24 compared to the placebo group (n = 35) (14.5% vs. 34.3% respectively, p = 0.02) and at week 48 (20.3% vs. 40%, p = 0.04). The unadjusted and adjusted annualized relapse rates were similar. The adjusted annualized relapse rate at week 24 was 0.37 for the rituximab group and 0.84 for the placebo group (p = 0.04) while at week 48 relapse rates were 0.37 and 0.72 (p = 0.08) respectively. Although study 4 [19] was not designed to examine relapse rate reduction authors found a relapse rate before treatment of 1.27 compared to 0.23 during treatment.

In terms of the radiological outcomes, three out of four studies included the number of Gad-enhancing lesions. For study 1 [16], the mean number of Gad-enhancing lesions was 1.31 at trial entry and decreased to 0.73 at week 4, 0.05 at week 48 and 0 at week 72. In study 2 [17], the mean number of Gad-enhancing lesions at weeks 12,16,20 and 24 was 5.5 in the placebo group and 0.5 in the rituximab group, with a reduction at each study time point beginning at week 12 (with p-values from 0.003 to <0.001). Considering the brain MRI scans obtained at weeks 12, 16, 20 and 24, 80.3% of the rituximab-treated subjects had no Gad-enhancing lesions compared to 51.4% in the placebo group. In study 3 [18], new Gad-enhancing lesions were not considered as contrast was not administered after baseline. In study 4 [19] 74% of the three post-treatment MRI scans were free of Gad-enhancing lesions. Mean number of Gad-enhancing lesions per month prior to treatment was 2.81 while after treatment it was 0.33.

The selected studies were heterogeneous and their primary and secondary outcomes as well as inclusion criteria were different. Not all studies were placebo-controlled and the characteristics of relapsing-remitting and primary-progressive patients are difficult to interpret together as the epidemiology and disease course vary for both. Considering all these facts, a meta-analysis was not considered appropriate for the scope of this systematic review.

## Discussion

This systematic review aimed to address the question of whether rituximab is efficacious and safe for use in MS patients. However, caution must be considered since there were several differences between the selected studies including the use of different outcomes, and different inclusion criteria (RRMS vs. PPMS).

Our systematic review addressed four different issues:

1 Is rituximab effective in reducing the time to confirmed disease progression (CDP)?

Only one of the studies used CDP as an outcome. Rituximab appeared to reduce progression by a marginal amount although the difference did not achieve statistical significance except in pre-planned analysis comparing young patients (under age 51 years) to older patients, and in those comparing patients with baseline Gad-enhancing lesions compared to those without baseline Gad-enhancing lesions. Another study explored disease progression comparing EDSS at the end of the study with baseline EDSS. Most patients (21/30) remained stable.

2 Is rituximab safe?

All the studies reported higher proportion of patients with AEs in the rituximab group compared to placebo with the first infusions. These infusion-related AEs decreased with subsequent infusions. There were three deaths in one of the studies (two in the placebo group and 1 in the rituximab) and in another study there was one death, none of them related to the drug (aspiration in a patient with history of brainstem lesions and the other one due to homicide). There were no grade 4 adverse events related to the infusions or infections. Most of the AEs that patients presented were classified as grade 1 or 2 (mild to moderate).

These results show that the use of rituximab for MS patients is accompanied by frequent but not serious adverse events that usually are reduced with subsequent infusions, and thus that rituximab is safe enough for these patients. However, the limited number of patients included in these studies and the relatively short duration of treatment and observation do not allow detecting long-term use-associated side effects. From the experience with the use of other monoclonal antibodies in MS, it has been observed that some adverse events such as PML appear more frequently after two full years of treatment. Thus, two year studies are likely to underestimate the true risk of this serious adverse event.

Rituximab has been widely used in other diseases such as rheumatologic or hematologic diseases typically combined with other drugs that affect the immune system. A study in the setting of rheumatoid arthritis described four patients from an estimated population of 129,000 exposed patients. Authors conclude that there is an increased risk of PML and they estimate the risk to be one per 25,000 treated individuals [20].

Another retrospective study evaluated the inclusion of rituximab in the treatment protocols for non-Hodgkin’s lymphomas. They found an increased risk of PML up to 2.2 per 1,000 patient-years. These analyses did not adjust the risk for concomitant treatment with broad spectrum immunosuppressive agents known to increase PML risk, so causality and true risk restricted to rituximab are difficult to establish [21].

The main adverse events described with the use of rituximab are infusion-related reactions. The risk of infections and hematological events (such as neutropenia) is markedly reduced compared to conventional chemotherapy used for non-Hodgkin lymphoma and chronic lymphocytic leukemia [22].

1. Does rituximab reduce the relapse rate?

Two of the four studies addressed this question. They showed that rituximab substantially reduced the relapse rate by week 24 and this reduction was observed also at week 48 (after one course of treatment) for one study and at week 72 for another (i.e. after 2 courses of therapy). Another study observed an important reduction also at week 52 although this study was not designed for relapse rate reduction.

The reduction in the relapse rate observed in patients receiving rituximab in study 2 [17] (56% at week 24 and at week 52) and in study 1 [16] (75% reduction from pre-therapy to week 24 and 87% from pre-therapy to week 48) was similar or greater than the one observed in the pivotal trials of natalizumab. Compared with placebo, natalizumab reduced relapse rate by 68% at one year and this effect was maintained at 2 years [23]. Monoclonal antibodies have a greater impact on the relapse rate than first-line disease-modifying therapies such as interferon beta (reductions of relapse rate between 32 and 34% at two years) [24], [25], [26] or glatiramer acetate (29% reduction at two years) [27].

When rituximab was used as an add-on therapy, relapse rate reduction was even greater (81% reduction at week 52) with the limitations that study 4 [19] was not designed to examine relapse rate reduction. In the pivotal trial combining natalizumab and interferon beta-1a vs. placebo plus interferon beta-1a, the reduction in the relapse rate was 54% [28], which was not greater than the effect seen in the monotherapy trial. As these trials were performed with different design and inclusion criteria, this does not allow for direct comparison of effectiveness.

1. Does rituximab reduce the number of Gad-enhancing lesions on brain MRI scans?

Three of four studies analyzed the effect of rituximab on the number of Gad-enhancing lesions on brain MRI scans. This number was reduced from 1.31, 2.1 or 2.84 depending on the study to 0.05 at week 48 and 0 at week 72 in one study, to 0 at week 24 and 48 in the second one and in the last one to 0.33. This means a reduction of 88% or greater when comparing the number of Gad-enhancing lesions before and after treatment or in the comparison between rituximab and placebo.

## Supporting Information

Table S1 **PRISMA Checklist.**
(DOC)
